# Supplementary material for: Immortalization of Different Breast Epithelial Cell Types Results in Distinct Mitochondrial Mutagenesis
Source: Int J Mol Sci. 2019 Jun 8;20(11):2813. doi: 10.3390/ijms20112813 (PMC6600575; doi:10.3390/ijms20112813)
Supplement: Supplementary file 1 [file ijms-20-02813-s001.pdf]

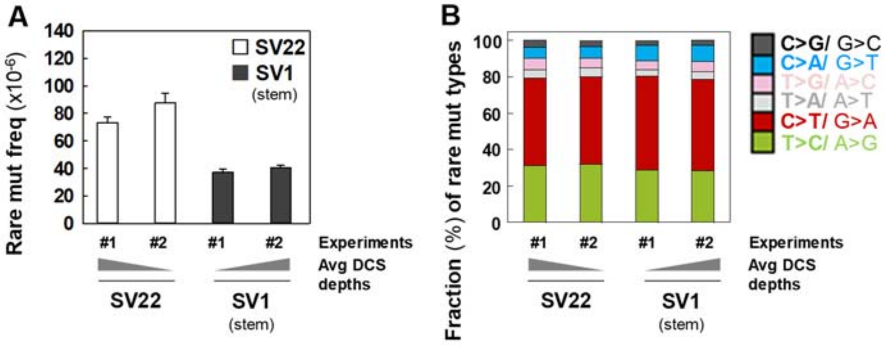

Figure S1. Reproducibility of Duplex Sequencing (DS) data: Frequencies and fractions (%) of rare mutations in the whole mtDNA. Data were from two independent DNA library experiments (Exp1 and Exp2) for DS. Overall rare mutation frequency (**A**) and fractions (%) of rare unique mutation types (**B**) were determined using DS for immortalized SV22 and SV1 cells. Variants that were mutated only once (singlets) were excluded. Error bars represent the Wilson Score 95% confidence intervals.

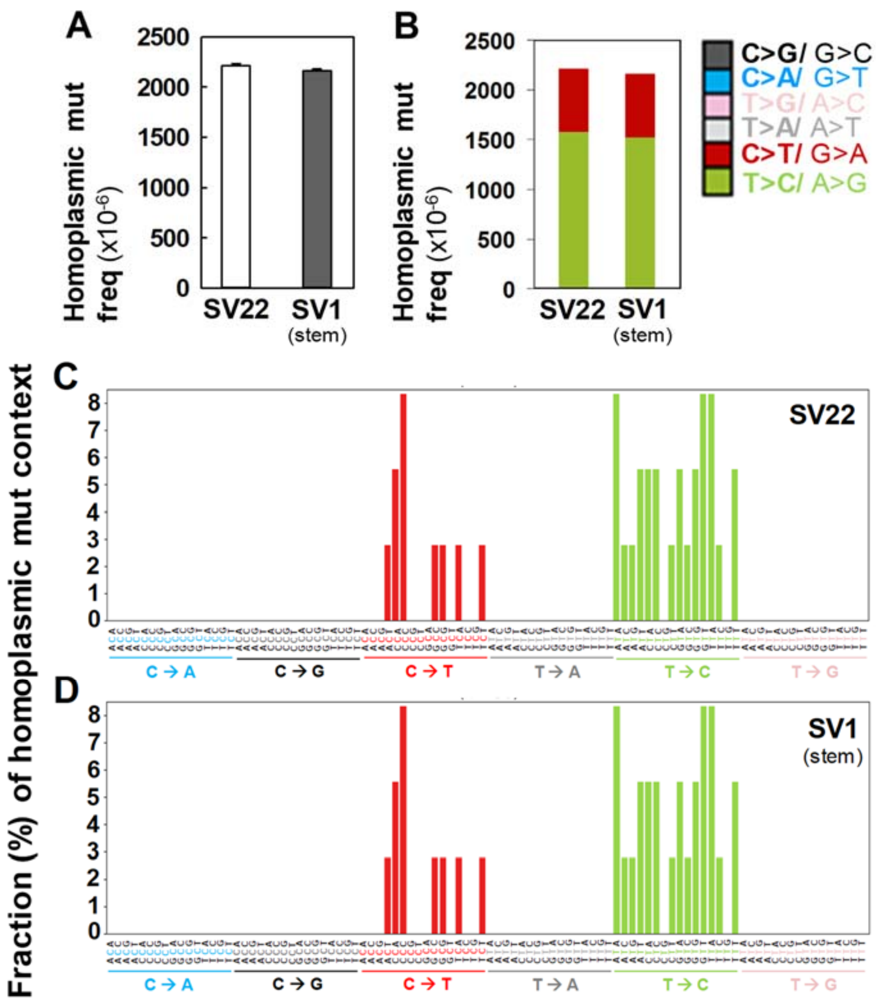

Figure S2. Frequencies and fractions (%) of sequence context spectra of homoplasmic mutations in the whole mtDNA. Overall homoplasmic mutation frequency (**A**), frequencies of each type of homoplasmic mutation (**B**), and homoplasmic unique mutation context spectra (**C,D**) for SV22 (immortalized non-stem) and SV1 (immortalized stem) cells were determined using Duplex Sequencing. Error bars represent the Wilson Score 95% confidence intervals.

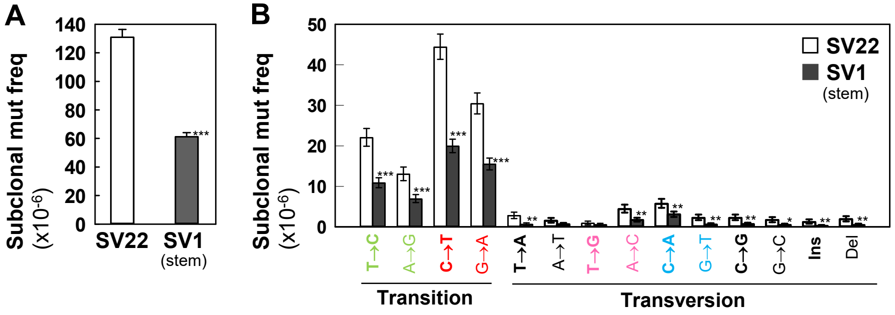

Figure S3. Frequencies of subclonal mutations in the whole mtDNA. Overall subclonal mutation frequency (**A**) and frequencies of each type of subclonal mutation (**B**) for SV22 (immortalized non-stem) and SV1 (immortalized stem) cells were determined using Duplex Sequencing. Error bars represent the Wilson Score 95% confidence intervals. Significant differences in subclonal mutation frequencies between two groups are indicated (* $p < 0.05$, ** $p < 5 \times 10^{-4}$, and *** $p < 5 \times 10^{-10}$) by the Chi-Square test.

A

	Fraction (%) of subclonal mut types					
	C>A/ G>T	C>G/ G>C	C>T/ G>A	T>A/ A>T	T>C/ A>G	T>G/ A>C
SV22	6	3	57	3.3	26.7	4
SV1 (stem)	5.9	1.8	57.7	2	29	3.6

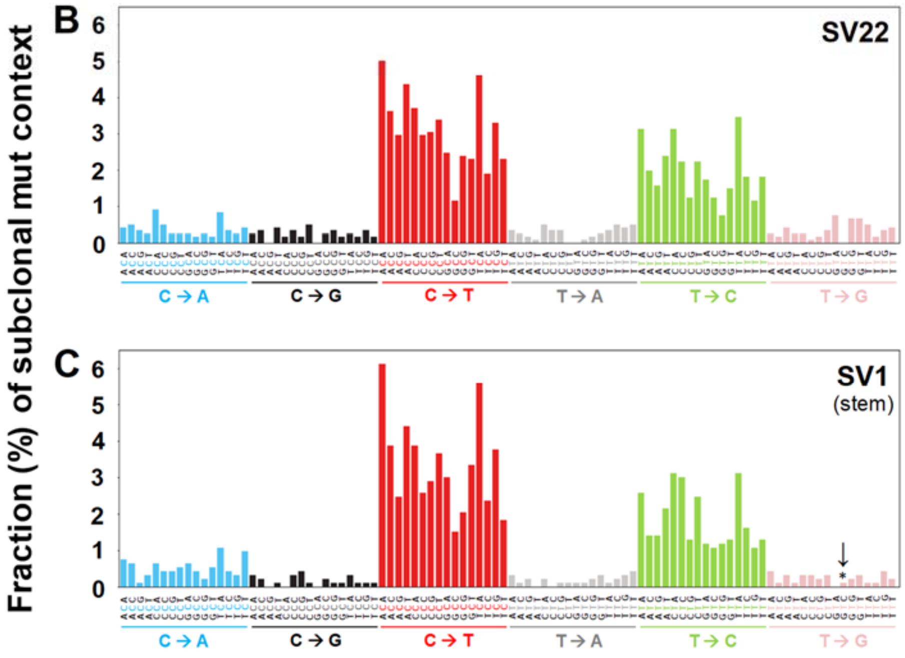

Figure S4. Types and sequence context spectra of subclonal unique mutations in the whole mtDNA. Fractions (%) of each type of subclonal unique mutation (**A**) and fractions (%) of subclonal mutation context spectra (**B**,**C**) for SV22 (immortalized non-stem) and SV1 (immortalized stem) cells were determined using Duplex Sequencing. Trinucleotide contexts (**B**,**C**) are the mutated base surrounded by all possibilities for its immediate 5' and 3' bases. To keep the graph concise, these point mutation trinucleotides are complemented as necessary to always depict the reference base as the pyrimidine of its pair. The fraction (%) of each specific trinucleotide out of all 96 possible trinucleotide contexts depicts the contribution of each genome sequence context to each point mutation type. Significant differences in fractions (%) of mutation context types between the two groups are indicated (* $p < 0.05$) by the 2-sample test for equality of proportions with continuity correction.

Table S1. The number of subclonal unique mutations in different regions of the whole mtDNA in immortalized human breast epithelial cells (SV22 and SV1). Only genome positions that had a DCS read depth of ≥100 in both samples were considered.

Subclonal unique mutations (0 to 10%)

		No. of subclonal unique muts	
		**SV22**	**SV1**
Protein coding region		735	677
Non-coding RNA	mt-RNR1 (rRNA12S)	175	87
	mt-RNR2 (rRNA16S)	187	114
	mt-tRNA	130	78
	Sum	492	279
Control region & overlapped		29	32
Sum		1256	988

Abbreviations used are: SV22, immortalized human breast epithelial cells (HBECs) derived from normal non-stem cells; SV1, immortalized HBECs derived from normal stem cells; DCS, duplex consensus sequence.

Table S2. Distribution of nonsynonymous unique mutations of subclonal variants within mtDNA coding regions in immortalized human breast epithelial cells (SV22 and SV1). Only genome positions that had a DCS read depth of ≥100 in both samples were considered.

A. Rare unique mutations (0 to 1%)

Cells	Whole mtDNA	Within mtDNA coding regions				Among unique mutations occurring within mtDNA coding regions	
	Total sequenced DCS Nts	No. of Syn	No. of Misse	No. of Nonsense	No. of Nonsyn	% Syn	% Nonsyn
SV22	17562378	511	207	9	216	70.3	29.7
SV1	27605473	463	196	12	208	69.0	31.0

B. Subclonal unique mutations (0 to 10%)

Cells	Whole mtDNA	Within mtDNA coding regions				Among unique mutations occurring within mtDNA coding regions	
	Total sequenced DCS Nts	No. of Syn.	No. of Misse	No. of Nonsense	No. of Nonsyn	% Syn	% Nonsyn
SV22	17562378	513	213	9	222	69.8	30.2
SV1	27605473	463	202	12	214	68.4	31.6

Abbreviations used are: Nts, nucleotides; DCS, duplex consensus sequences; mt, mitochondrial; Nonsyn, nonsynonymous mutation; Misse, missense mutation; Syn, synonymous mutation. SV22, immortalized human breast epithelial cells (HBECs) derived from normal non-stem cells; SV1, immortalized HBECs derived from normal stem cells. The numbers of nonsynonymous mutations are sums of missense and nonsense (truncating) mutations.

Table S3. Number of unique mutations identified in the whole mtDNA of immortalized human breast epithelial cells (SV22 and SV1) using Duplex Sequencing. Only genome positions that had a DCS read depth of ≥100 in both samples were considered.

	No. of unique mutations		
	0-1%	0-10%	0-100%
SV22 only [a]	792	797	797
SV1 only [b]	527	528	528
Both SV22 and SV1 [c]	447	459	501
SV22 ↑3X > SV1 [d]	88	92	93
SV22 < SV1 ↑3X [e]	9	11	11

Abbreviations used are:
SV22, immortalized human breast epithelial cells (HBECs) derived from normal non-stem cells; SV1, immortalized HBECs derived from normal stem cells; DCS, duplex consensus sequence.

[a] Mutations found only in SV22 cells but not in SV1 cells
[b] Mutations found only in SV1 cells but not in SV22 cells
[c] Mutations found in both SV22 and SV1 cells
[d] Mutations found in both SV22 and SV1 cells: With being more highly mutated at ≧ 3-fold in SV22 than in SV1 cells
[e] Mutations found in both SV22 and SV1 cells: With being more highly mutated at ≧ 3-fold in SV1 than in SV22 cells

Table S4. Mutations identified using Duplex Sequencing in the whole mtDNA of immortalized human breast epithelial cells (SV22 and SV1).

Abbreviations used are: mt, mitochondrial; Nonsyn, nonsynonymous mutation; Syn, synonymous mutation. SV22, immortalized human breast epithelial cells (HBECs) derived from normal non-stem cells; SV1, immortalized HBECs derived from normal stem cells.

A. Subclonal variants (excluding singlets) found only in SV22 cells, but not in SV1 cells
Among the mutations identified, variants that have mutated at least twice in each position are listed below.

Mt Gene	Mutation type	Amino acid change		SV22 allele frequency (%)	g-score	mitoTIP predicted score
Control region	C>A	.	Non-coding	0.20	.	.
Control region	A>G	.	Non-coding	0.36	.	.
Control region	A>G	.	Non-coding	2.06	.	.
Control region	C>T	.	Non-coding	0.16	.	.
MT-RNR1	G>A	.	ncRNA	0.17	.	.
MT-RNR1	C>G	.	ncRNA	0.19	.	.
MT-RNR1	G>C	.	ncRNA	0.19	.	.
MT-RNR1	T>C	.	ncRNA	0.19	.	.
MT-RNR1	G>A	.	ncRNA	0.20	.	.
MT-RNR1	A>G	.	ncRNA	0.20	.	.
MT-RNR1	C>T	.	ncRNA	0.20	.	.
MT-RNR1	T>C	.	ncRNA	0.20	.	.
MT-RNR1	C>T	.	ncRNA	0.20	.	.
MT-RNR1	G>A	.	ncRNA	0.20	.	.
MT-RNR1	A>G	.	ncRNA	0.21	.	.
MT-RNR1	G>A	.	ncRNA	0.21	.	.
MT-RNR1	C>A	.	ncRNA	0.22	.	.
MT-RNR1	C>A	.	ncRNA	0.23	.	.
MT-RNR1	A>G	.	ncRNA	0.25	.	.
MT-RNR1	G>A	.	ncRNA	0.27	.	.
MT-RNR1	A>C	.	ncRNA	0.27	.	.
MT-RNR1	T>G	.	ncRNA	0.29	.	.
MT-RNR1	C>T	.	ncRNA	0.29	.	.
MT-RNR1	G>T	.	ncRNA	0.31	.	.
MT-RNR1	C>T	.	ncRNA	0.33	.	.
MT-RNR1	C>T	.	ncRNA	0.34	.	.
MT-RNR1	C>T	.	ncRNA	0.34	.	.
MT-RNR1	C>T	.	ncRNA	0.35	.	.
MT-RNR1	T>C	.	ncRNA	0.35	.	.
MT-RNR1	T>C	.	ncRNA	0.37	.	.
MT-RNR1	C>T	.	ncRNA	0.38	.	.
MT-RNR1	C>T	.	ncRNA	0.39	.	.
MT-RNR1	C>G	.	ncRNA	0.47	.	.
MT-RNR1	G>A	.	ncRNA	0.51	.	.
MT-RNR1	G>A	.	ncRNA	0.55	.	.

MT-RNR1	G>A	.	ncRNA	0.55	.	.
MT-RNR1	C>T	.	ncRNA	0.70	.	.
MT-RNR1	C>T	.	ncRNA	0.73	.	.
MT-RNR1	C>A	.	ncRNA	1.12	.	.
MT-TV	A>G	.	ncRNA	0.18	.	16.7
MT-TV	T>C	.	ncRNA	0.20	.	17.0
MT-RNR2	C>T	.	ncRNA	0.15	.	.
MT-RNR2	T>C	.	ncRNA	0.16	.	.
MT-RNR2	T>A	.	ncRNA	0.16	.	.
MT-RNR2	A>T	.	ncRNA	0.18	.	.
MT-RNR2	A>C	.	ncRNA	0.18	.	.
MT-RNR2	T>A	.	ncRNA	0.18	.	.
MT-RNR2	G>T	.	ncRNA	0.18	.	.
MT-RNR2	A>C	.	ncRNA	0.18	.	.
MT-RNR2	C>T	.	ncRNA	0.19	.	.
MT-RNR2	A>G	.	ncRNA	0.19	.	.
MT-RNR2	G>A	.	ncRNA	0.19	.	.
MT-RNR2	C>T	.	ncRNA	0.20	.	.
MT-RNR2	G>A	.	ncRNA	0.20	.	.
MT-RNR2	T>C	.	ncRNA	0.21	.	.
MT-RNR2	G>A	.	ncRNA	0.23	.	.
MT-RNR2	T>C	.	ncRNA	0.23	.	.
MT-RNR2	A>G	.	ncRNA	0.23	.	.
MT-RNR2	G>A	.	ncRNA	0.27	.	.
MT-RNR2	G>A	.	ncRNA	0.27	.	.
MT-RNR2	G>A	.	ncRNA	0.27	.	.
MT-RNR2	T>C	.	ncRNA	0.27	.	.
MT-RNR2	T>C	.	ncRNA	0.27	.	.
MT-RNR2	C>T	.	ncRNA	0.28	.	.
MT-RNR2	G>A	.	ncRNA	0.28	.	.
MT-RNR2	C>T	.	ncRNA	0.32	.	.
MT-RNR2	T>C	.	ncRNA	0.33	.	.
MT-RNR2	C>T	.	ncRNA	0.35	.	.
MT-RNR2	T>C	.	ncRNA	0.36	.	.
MT-RNR2	G>A	.	ncRNA	0.36	.	.
MT-RNR2	A>G	.	ncRNA	0.38	.	.
MT-RNR2	G>A	.	ncRNA	0.39	.	.
MT-RNR2	C>T	.	ncRNA	0.45	.	.
MT-RNR2	G>A	.	ncRNA	0.48	.	.
MT-RNR2	C>T	.	ncRNA	0.54	.	.
MT-RNR2	C>T	.	ncRNA	0.54	.	.
MT-RNR2	T>C	.	ncRNA	0.58	.	.
MT-RNR2	C>T	.	ncRNA	0.59	.	.
MT-RNR2	C>T	.	ncRNA	0.69	.	.
MT-ND1	A>C	Missense	T87P	0.17	0.22	.

MT-ND2	C>T	Syn	P138P	0.27	.	.
MT-ND2	G>A	Missense	G188E	0.44	0.87	.
MT-ND2	C>T	Missense	T226M	0.17	0.25	.
MT-ND2	C>T	Syn	T258T	0.28	.	.
MT-ND2	G>A	Syn	Q316Q	1.17	.	.
MT-ND2	C>T	Syn	T333T	0.88	.	.
MT-TW	C>T	.	ncRNA	0.21	.	3.1
MT-TW	A>G	.	ncRNA	0.21	.	1.2
MT-TW	C>T	.	ncRNA	0.45	.	15.5
MT-TA	T>C	.	ncRNA	0.20	.	4.4
MT-TA	T>C	.	ncRNA	0.20	.	16.7
MT-TA	C>T	.	ncRNA	0.20	.	0.9
MT-TA	C>A	.	ncRNA	0.20	.	4.7
MT-COX1	C>T	Syn	Y54Y	0.23	.	.
MT-COX1	A>G	Syn	W81W	0.14	.	.
MT-COX1	A>G	Syn	W126W	0.14	.	.
MT-COX1	C>T	Syn	H290H	0.15	.	.
MT-COX1	T>C	Syn	F293F	0.17	.	.
MT-COX1	C>T	Syn	D298D	0.16	.	.
MT-COX1	C>T	Syn	I312I	0.16	.	.
MT-COX1	C>T	Syn	P315P	0.17	.	.
MT-COX1	C>A	Nonsense	S362X	0.17	.	.
MT-COX1	C>T	Syn	H376H	0.22	.	.
MT-COX1	T>G	Syn	A385A	0.17	.	.
MT-COX1	C>T	Syn	Y403Y	0.38	.	.
MT-COX1	T>C	Syn	T424T	0.43	.	.
MT-COX1	C>T	Syn	I419I	0.55	.	.
MT-COX2	C>A	Missense	F36L	1.66	0.12	.
MT-COX2	G>A	Missense	V38I	0.36	0.18	.
MT-COX2	C>T	Missense	L84F	0.20	0.65	.
MT-COX2	C>T	Missense	L144F	3.32	0.68	.
MT-COX2	C>T	Missense	T226I	0.80	0.12	.
MT-TK	G>A	.	ncRNA	0.41	.	17.5
MT-ATP6	T>C	Syn	Y36Y	0.21	.	.
MT-ATP6	C>T	Syn	I43I	0.19	.	.
MT-ATP6	C>T	Missense	R66W	0.56	0.18	.
MT-ATP6	G>A	Syn	L156L	0.27	.	.
MT-ATP8	T>C	Syn	L29L	0.19	.	.
MT-COX3	G>A	Syn	M33M	0.18	.	.
MT-COX3	C>T	Syn	H115H	0.48	.	.
MT-ND3	G>T	Nonsense	E105X	0.77	.	.
MT-ND4	C>T	Missense	S101F	0.16	0.11	.
MT-ND4	A>C	Syn	L102L	0.24	.	.
MT-ND4	T>A	Missense	I104N	0.16	0.44	.
MT-ND4	C>T	Syn	I116I	0.16	.	.

MT-ND4	T>C	Syn	F118F	0.16	.	.
MT-ND4	T>C	Syn	Y119Y	0.17	.	.
MT-ND4	G>A	Missense	A287T	0.84	0.32	.
MT-ND4	G>A	Syn	L376L	0.19	.	.
MT-ND4	G>T	Syn	L376L	0.66	.	.
MT-ND4	G>A	Syn	T385T	0.17	.	.
MT-ND4	A>G	Syn	S418S	0.19	.	.
MT-ND4	C>G	Missense	T420S	0.18	0.29	.
MT-TS2	T>C	.	ncRNA	0.18	.	2.9
MT-ND5	T>A	Nonsense	Y35X	0.19	.	.
MT-ND5	G>A	Missense	V40I	0.27	0.06	.
MT-ND5	C>T	Syn	Y84Y	0.17	.	.
MT-ND5	C>T	Missense	L216F	0.25	0.21	.
MT-ND5	C>T	Syn	L229L	0.17	.	.
MT-ND5	C>T	Syn	L266L	0.21	.	.
MT-ND5	G>A	Missense	S270N	0.18	0.04	.
MT-ND5	A>C	Syn	L276L	0.28	.	.
MT-ND5	T>C	Syn	L280L	0.45	.	.
MT-ND5	T>C	Missense	I283T	0.19	0.36	.
MT-ND5	T>C	Syn	T285T	0.29	.	.
MT-ND5	T>C	Syn	I566I	0.18	.	.
MT-ND5	A>G	Missense	T573A	0.18	0.04	.
MT-ND5	C>T	Syn	T579T	0.16	.	.
MT-ND5	A>C	Missense	I596L	0.18	0.07	.
MT-ND5	T>C	Syn	I584I	0.21	.	.
MT-ND6	A>T	Missense	F46Y	0.29	0.50	.
MT-ND6	C>T	Syn	M51M	0.20	.	.
MT-ND6	T>C	Syn	M54M	0.40	.	.
MT-ND6	T>A	Syn	G62G	0.21	.	.
MT-ND6	C>T	Syn	M63M	0.38	.	.
MT-ND6	A>G	Syn	V65V	0.18	.	.
MT-ND6	G>A	Syn	V66V	0.48	.	.
MT-ND6	A>G	Syn	Y69Y	0.20	.	.
MT-ND6	C>T	Syn	A72A	0.19	.	.
MT-ND6	C>T	Syn	M73M	0.29	.	.
MT-ND6	C>T	Syn	V90V	0.22	.	.
MT-ND6	T>C	Syn	K107K	0.20	.	.
MT-ND6	A>T	Syn	V114V	0.19	.	.
MT-ND6	C>T	Missense	V114I	0.20	0.22	.
MT-ND6	G>A	Syn	N117N	0.30	.	.
MT-ND6	T>C	Syn	G129G	0.17	.	.
MT-ND6	C>G	Missense	G133A	0.16	0.79	.
MT-ND6	C>T	Syn	G133G	0.16	.	.
MT-ND6	A>G	Missense	S132P	0.24	0.67	.
MT-ND6	T>C	Syn	V121V	0.29	.	.

MT-CYTB	C>A	Missense	H16Q	0.15	0.20	.
MT-CYTB	T>C	Missense	F18S	0.30	0.39	.
MT-CYTB	G>A	Missense	A29T	0.16	0.09	.
MT-CYTB	T>C	Missense	F33L	0.16	0.30	.
MT-CYTB	C>T	Missense	S110L	0.17	0.14	.
MT-CYTB	T>C	Missense	S110P	0.17	0.43	.
MT-CYTB	T>A	Nonsense	S172X	0.15	.	.
MT-CYTB	T>C	Syn	F181F	0.16	.	.
MT-CYTB	C>T	Syn	Y224Y	0.84	.	.
MT-CYTB	A>G	Syn	L250L	0.18	.	.
MT-CYTB	G>A	Missense	G251D	0.18	0.36	.
MT-CYTB	T>C	Syn	L262L	0.28	.	.
MT-CYTB	G>A	Missense	R318H	0.28	0.08	.
MT-CYTB	C>T	Syn	L320L	0.29	.	.
MT-CYTB	A>C	Syn	S323S	0.18	.	.
MT-CYTB	C>T	Missense	S323L	0.18	0.03	.
MT-CYTB	T>A	Syn	L324L	0.18	.	.
MT-CYTB	C>T	Missense	L327F	0.19	0.09	.
MT-CYTB	G>A	Missense	A330T	0.20	0.09	.
MT-TT	G>A	.	ncRNA	0.19	.	2.4

B. Subclonal variants (excluding singlets) found only in SV1 cells, but not in SV22 cells
Among the mutations identified, variants that have mutated at least twice in each position are listed below.

Mt Gene	Mutation type	Amino acid change		SV1 allele frequency (%)	g-score	mitoTIP predicted score
Control region	G>C	.	Non-coding	1.69	.	.
Control region	C>T	.	Non-coding	0.47	.	.
MT-RNR1	T>C	.	ncRNA	0.09	.	.
MT-RNR1	G>A	.	ncRNA	0.09	.	.
MT-RNR1	C>T	.	ncRNA	0.08	.	.
MT-RNR2	A>G	.	ncRNA	0.11	.	.
MT-RNR2	G>A	.	ncRNA	0.11	.	.
MT-RNR2	G>C	.	ncRNA	0.13	.	.
MT-RNR2	T>C	.	ncRNA	0.11	.	.
MT-RNR2	T>C	.	ncRNA	0.10	.	.
MT-RNR2	C>T	.	ncRNA	0.15	.	.
MT-RNR2	G>A	.	ncRNA	0.16	.	.
MT-RNR2	C>T	.	ncRNA	0.10	.	.
MT-RNR2	A>G	.	ncRNA	0.10	.	.
MT-RNR2	C>T	.	ncRNA	0.19	.	.
MT-ND1	T>C	Missense	F56S	0.14	0.69	.
MT-ND1	A>C	Missense	T57P	0.23	0.22	.
MT-ND1	G>C	Missense	E59D	0.16	0.21	.
MT-ND1	T>A	Missense	L61Q	0.61	0.47	.

MT-ND1	A>C	Missense	K62N	0.36	0.16	.
MT-ND1	G>A	Syn	L176L	0.10	.	.
MT-ND1	T>C	Missense	Y304H	0.36	0.09	.
MT-TN	A>C	.	ncRNA	0.22	.	8.3
MT-COX1	G>A	Syn	E40E	0.16	.	.
MT-COX1	A>G	Syn	V70V	0.13	.	.
MT-COX1	C>T	Syn	H151H	0.12	.	.
MT-COX1	G>A	Missense	V155I	0.28	0.06	.
MT-COX1	A>C	Missense	K172N	0.28	0.39	.
MT-COX1	C>A	Syn	G250G	0.08	.	.
MT-COX1	C>T	Syn	Y260Y	0.08	.	.
MT-COX1	T>C	Missense	S262P	0.08	0.59	.
MT-COX1	A>G	Syn	K265K	0.07	.	.
MT-COX1	T>C	Syn	G272G	0.07	.	.
MT-COX1	G>A	Syn	G284G	0.11	.	.
MT-COX1	A>T	Syn	A303A	0.08	.	.
MT-COX1	G>A	Missense	A313T	0.08	0.47	.
MT-COX1	C>T	Syn	S322S	0.08	.	.
MT-COX1	A>G	Syn	W323W	0.08	.	.
MT-COX1	A>G	Syn	A337A	0.08	.	.
MT-COX1	A>G	Syn	L342L	0.12	.	.
MT-COX1	A>G	Syn	V350V	0.13	.	.
MT-COX2	C>T	Syn	Y105Y	0.09	.	.
MT-COX2	C>T	Missense	P125L	0.16	0.15	.
MT-COX2	G>A	Syn	L160L	0.23	.	.
MT-COX2	G>A	Syn	P189P	0.19	.	.
MT-COX2	T>C	Syn	G194G	0.19	.	.
MT-ATP8	C>A	Missense	P53Q	0.17	0.16	.
MT-COX3	T>C	Syn	L137L	0.09	.	.
MT-COX3	T>C	Syn	A147A	0.08	.	.
MT-COX3	A>C	Syn	V142V	0.09	.	.
MT-COX3	C>T	Syn	T145T	0.09	.	.
MT-COX3	G>A	Syn	L169L	0.12	.	.
MT-COX3	C>G	Missense	L171V	0.12	0.21	.
MT-COX3	C>T	Syn	L171L	0.12	.	.
MT-COX3	T>C	Syn	Y172Y	0.12	.	.
MT-COX3	C>T	Syn	Y181Y	0.13	.	.
MT-ND3	A>C	Missense	T35P	0.12	0.49	.
MT-ND3	A>C	Missense	T61P	0.10	0.86	.
MT-ND3	C>T	Syn	L86L	0.09	.	.
MT-ND3	T>C	Syn	V88V	0.09	.	.
MT-ND3	G>A	Syn	M89M	0.09	.	.
MT-ND3	T>C	Missense	M89T	0.09	0.07	.
MT-ND3	A>G	Syn	S90S	0.09	.	.
MT-ND3	T>C	Syn	Y104Y	0.09	.	.

MT-ND3	G>A	Syn	E105E	0.09	.	.
MT-ND3	C>T	Syn	L107L	0.09	.	.
MT-ND3	C>T	Nonsense	Q108X	0.09	.	.
MT-ND3	A>G	Syn	G110G	0.13	.	.
MT-TR	T>C	.	ncRNA	0.17	.	17.8
MT-ND4L	G>A	Syn	M1M	0.09	.	.
MT-ND4L	T>C	Syn	A44A	0.14	.	.
MT-ND4	T>C	Syn	I9I	0.19	.	.
MT-ND4	G>A	Syn	L14L	0.15	.	.
MT-ND4	C>T	Missense	A131V	0.10	0.50	.
MT-ND4	T>A	Syn	A198A	0.11	.	.
MT-ND4	T>C	Syn	F203F	0.11	.	.
MT-ND4	G>A	Syn	K206K	0.17	.	.
MT-ND4	A>G	Syn	M207M	0.12	.	.
MT-ND4	T>C	Syn	P208P	0.12	.	.
MT-ND4	T>C	Syn	L209L	0.12	.	.
MT-ND4	G>A	Nonsense	G211X	0.12	.	.
MT-ND4	T>C	Syn	L214L	0.24	.	.
MT-ND4	A>G	Syn	W215W	0.12	.	.
MT-ND4	A>G	Syn	V234V	0.14	.	.
MT-ND4	C>T	Syn	G239G	0.15	.	.
MT-ND4	T>C	Syn	G242G	0.15	.	.
MT-ND4	A>G	Syn	M244M	0.15	.	.
MT-ND4	A>C	Syn	T247T	0.16	.	.
MT-ND4	T>C	Syn	T337T	0.12	.	.
MT-ND4	C>T	Syn	N425N	0.41	.	.
MT-ND4	G>A	Syn	M437M	0.11	.	.
MT-ND4	T>C	Syn	I444I	0.11	.	.
MT-ND4	C>T	Syn	L449L	0.11	.	.
MT-ND4	C>T	Syn	D452D	0.11	.	.
MT-ND4	T>C	Syn	S459S	0.12	.	.
MT-ND4	C>T	Syn	P451P	0.11	.	.
MT-ND5	C>T	Missense	H4Y	0.12	0.12	.
MT-ND5	A>C	Missense	T8P	0.18	0.28	.
MT-ND5	A>G	Missense	T11A	0.18	0.11	.
MT-ND5	C>T	Syn	L15L	0.18	.	.
MT-ND5	C>T	Syn	P18P	0.18	.	.
MT-ND5	G>A	Missense	S47N	0.51	0.28	.
MT-ND5	C>T	Syn	I123I	0.11	.	.
MT-ND5	C>A	Syn	P240P	0.27	.	.
MT-ND5	C>T	Syn	V243V	0.08	.	.
MT-ND5	C>T	Syn	A245A	0.08	.	.
MT-ND5	C>T	Missense	A245V	0.08	0.33	.
MT-ND5	G>A	Missense	S377N	0.12	0.09	.
MT-ND5	A>G	Syn	L380L	0.13	.	.

MT-ND5	G>A	Missense	A399T	0.14	0.16	.
MT-ND5	A>C	Syn	T404T	0.13	.	.
MT-ND5	T>C	Syn	A415A	0.12	.	.
MT-ND5	C>T	Missense	S473F	0.21	0.20	.
MT-CYTB	G>A	Syn	M53M	0.16	.	.
MT-CYTB	C>T	Syn	A59A	0.15	.	.
MT-CYTB	A>G	Syn	S65S	0.10	.	.
MT-CYTB	C>T	Syn	H68H	0.14	.	.
MT-CYTB	G>A	Syn	P134P	0.15	.	.
MT-CYTB	A>C	Syn	S139S	0.23	.	.
MT-CYTB	C>A	Missense	I211M	0.12	0.12	.
MT-CYTB	G>A	Missense	S344N	0.17	0.26	.
MT-CYTB	C>T	Syn	T348T	0.17	.	.

C. Subclonal variants found in both SV22 and SV1 cells: With being more highly mutated at ≧ 3-fold in SV22 than in SV1 cells

Mt Gene	DNA mutation	Amino acid change		SV22 allele frequency (%)	SV1 allele frequency (%)	Fold Change (SV22/SV1)	g-score	mitoTIP predicted score
Control region	G>C	.	Non-coding	0.29	0.04	6.62	.	.
Control region	C>G	.	Non-coding	0.19	0.04	4.85	.	.
MT-RNR1	A>G	.	ncRNA	0.26	0.06	4.42	.	.
MT-RNR1	T>C	.	ncRNA	0.31	0.05	5.71	.	.
MT-RNR1	A>G	.	ncRNA	0.30	0.05	6.01	.	.
MT-RNR1	T>A	.	ncRNA	0.52	0.06	9.30	.	.
MT-RNR1	C>T	.	ncRNA	0.21	0.07	3.21	.	.
MT-RNR1	G>A	.	ncRNA	0.75	0.19	4.00	.	.
MT-RNR1	G>A	.	ncRNA	0.45	0.10	4.41	.	.
MT-RNR1	C>G	.	ncRNA	0.49	0.07	6.75	.	.
MT-RNR1	A>G	.	ncRNA	0.21	0.07	3.10	.	.
MT-RNR1	C>T	.	ncRNA	0.43	0.12	3.46	.	.
MT-RNR1	G>A	.	ncRNA	0.87	0.17	5.08	.	.
MT-RNR1	C>A	.	ncRNA	0.89	0.17	5.26	.	.
MT-RNR1	C>T	.	ncRNA	1.20	0.20	6.04	.	.
MT-RNR1	G>A	.	ncRNA	0.19	0.05	3.95	.	.
MT-RNR1	C>T	.	ncRNA	1.18	0.10	11.41	.	.
MT-RNR1	G>A	.	ncRNA	0.42	0.05	8.04	.	.
MT-RNR1	G>A	.	ncRNA	0.89	0.15	5.84	.	.
MT-RNR1	A>T	.	ncRNA	0.21	0.05	4.28	.	.
MT-RNR1	T>C	.	ncRNA	0.46	0.05	9.71	.	.
MT-RNR1	G>A	.	ncRNA	0.23	0.04	5.12	.	.
MT-RNR1	G>T	.	ncRNA	0.46	0.04	10.24	.	.
MT-RNR1	T>C	.	ncRNA	0.54	0.05	11.84	.	.

MT-RNR1	C>T	.	ncRNA	0.40	0.04	9.35	.	.
MT-RNR1	T>C	.	ncRNA	0.22	0.04	5.05	.	.
MT-RNR1	G>C	.	ncRNA	0.22	0.05	4.76	.	.
MT-RNR1	T>A	.	ncRNA	0.21	0.05	4.32	.	.
MT-RNR1	G>A	.	ncRNA	1.26	0.42	3.02	.	.
MT-RNR2	A>G	.	ncRNA	0.34	0.10	3.47	.	.
MT-RNR2	G>A	.	ncRNA	0.45	0.05	8.32	.	.
MT-RNR2	C>T	.	ncRNA	0.27	0.06	4.11	.	.
MT-RNR2	G>A	.	ncRNA	0.30	0.06	4.89	.	.
MT-RNR2	C>T	.	ncRNA	0.28	0.07	3.91	.	.
MT-RNR2	T>C	.	ncRNA	0.38	0.07	5.25	.	.
MT-RNR2	C>T	.	ncRNA	0.30	0.07	4.04	.	.
MT-RNR2	T>C	.	ncRNA	0.30	0.07	4.15	.	.
MT-RNR2	T>C	.	ncRNA	0.45	0.09	4.90	.	.
MT-RNR2	C>T	.	ncRNA	0.47	0.10	4.60	.	.
MT-RNR2	G>A	.	ncRNA	9.63	1.53	6.30	.	.
MT-RNR2	G>A	.	ncRNA	0.50	0.07	7.20	.	.
MT-TL1	G>A	.	ncRNA	0.32	0.05	5.82	.	2.1
MT-TL1	T>C	.	ncRNA	0.37	0.05	6.97	.	2.0
MT-TL1	C>T	.	ncRNA	0.31	0.05	5.76	.	1.5
MT-ND2	A>G	Syn	M163M	0.30	0.05	5.84	.	.
MT-ND2	G>A	Missense	Q174Q	0.55	0.15	3.58	.	.
MT-ND2	T>C	Syn	M191T	0.21	0.04	4.93	0.22	.
MT-TA	C>T	.	ncRNA	0.46	0.04	11.78	.	6.3
MT-TA	C>T	.	ncRNA	0.41	0.04	9.98	.	12.3
MT-TA	G>A	.	ncRNA	0.45	0.08	5.79	.	12.0
MT-TN	C>T	.	ncRNA	0.36	0.04	9.15	.	2.4
MT-TN	G>A	.	ncRNA	0.74	0.21	3.59	.	4.0
MT-TN	C>T	.	ncRNA	0.37	0.08	4.58	.	10.4
MT-TN	C>G	.	ncRNA	0.17	0.04	4.44	.	18.1
MT-TC	C>T	.	ncRNA	0.18	0.04	4.88	.	13.6
MT-TC	T>A	.	ncRNA	0.36	0.08	4.75	.	1.2
MT-TC	T>C	.	ncRNA	0.17	0.04	4.61	.	2.5
MT-TC	C>T	.	ncRNA	0.27	0.08	3.49	.	-1.7
MT-COX1	C>T	Syn	L183L	0.20	0.04	4.71	.	.
MT-COX1	C>T	Syn	F238F	0.25	0.07	3.43	.	.
MT-COX1	T>C	Syn	G239G	0.17	0.04	4.50	.	.
MT-COX1	T>C	Syn	P241P	0.17	0.04	4.69	.	.
MT-COX1	T>C	Syn	V243V	0.17	0.04	4.61	.	.
MT-COX1	C>T	Syn	F251F	0.19	0.04	4.82	.	.

MT-COX1	A>G	Syn	W275W	0.16	0.04	4.45	.	.
MT-COX1	G>A	Syn	M277M	0.24	0.04	6.76	.	.
MT-COX1	A>C	Syn	A289A	0.23	0.04	6.21	.	.
MT-COX1	T>C	Syn	Y304Y	0.25	0.04	6.03	.	.
MT-COX1	C>T	Syn	L363L	0.33	0.11	3.12	.	.
MT-COX1	G>A	Syn	T370T	0.40	0.11	3.64	.	.
MT-COX1	T>C	Syn	V373V	0.39	0.11	3.50	.	.
MT-COX1	A>G	Syn	L399L	0.31	0.06	5.42	.	.
MT-COX1	C>T	Syn	A410A	0.20	0.06	3.15	.	.
MT-COX1	C>T	Syn	F414F	0.68	0.06	11.02	.	.
MT-COX1	T>C	Syn	N422N	0.86	0.07	12.71	.	.
MT-COX1	C>T	Syn	L431L	0.37	0.07	5.23	.	.
MT-COX1	C>T	Syn	S434S	0.46	0.07	6.37	.	.
MT-COX1	C>T	Syn	N451N	0.60	0.16	3.67	.	.
MT-ATP6	A>G	Missense	M1V	0.31	0.08	3.95	0.76	.
MT-ATP8	A>G	Syn	K54K	0.31	0.08	3.95	.	.
MT-COX3	T>C	Missense	M40T	0.31	0.10	3.13	0.07	.
MT-ND5	C>T	Syn	Y32Y	0.18	0.05	3.27	.	.
MT-ND5	G>A	Missense	Q116Q	0.17	0.06	3.00	.	.
MT-ND5	C>T	Syn	A225A	0.25	0.04	5.89	.	.
MT-ND5	T>A	Syn	L246Q	0.19	0.04	4.58	0.60	.
MT-ND6	T>C	Syn	G68G	0.39	0.07	5.95	.	.
MT-ND6	G>A	Syn	S123S	0.36	0.06	6.35	.	.
MT-CYTB	C>T	Syn	L36L	0.25	0.05	5.27	.	.
MT-CYTB	G>A	Syn	L41L	0.19	0.05	3.72	.	.
MT-CYTB	T>C	Syn	N74N	0.27	0.04	6.15	.	.
MT-CYTB	G>C	Syn	G99G	0.27	0.05	5.09	.	.
MT-CYTB	A>C	Syn	G105G	0.18	0.06	3.12	.	.

D. Subclonal variants found in both SV22 and SV1 cells: With being more highly mutated at ≥ 3- fold in SV1 than in SV22 cells

Mt Gene	DNA mutation	Amino acid change		SV22 allele frequency (%)	SV1 allele frequency (%)	Fold Change (SV1/SV22)	g-score	mitoTIP predicted score
MT-TY	C>T	.	ncRNA	0.08	0.38	4.98	.	5.0
MT-COX1	C>T	Syn	A325A	0.08	0.44	5.47	.	.
MT-TD	G>A	.	ncRNA	0.16	0.74	4.65	.	0.71
MT-COX2	G>A	Syn	E109E	0.08	0.32	3.88	.	.
MT-ATP6	G>C	Missense	A11P	0.32	1.44	4.43	0.27	.
MT-ATP8	G>C	Missense	L64F	0.32	1.44	4.43	0.12	.
MT-COX3	C>T	Syn	A107A	0.09	0.30	3.25	.	.
MT-COX3	T>C	Syn	P108P	0.10	0.31	3.15	.	.

MT-COX3	T>C	Syn	L112L	0.10	0.40	4.01	.	.
MT-COX3	A>G	Syn	G113G	0.10	0.35	3.46	.	.
MT-COX3	G>A	Syn	G114G	0.10	0.39	4.03	.	.
